# Supplementary material for: Single-cell transcriptional profiling of human thymic stroma uncovers novel cellular heterogeneity in the thymic medulla
Source: Nat Commun. 2021 Feb 17;12:1096. doi: 10.1038/s41467-021-21346-6 (PMC7889611; doi:10.1038/s41467-021-21346-6)
Supplement: Supplementary file 1 — Supplementary Information [file 41467_2021_21346_MOESM1_ESM.pdf]

Supplementary Information

Single-cell transcriptional profiling of human thymic stroma uncovers novel cellular heterogeneity in the thymic medulla

Jhoanne L. Bautista, Nathan T. Cramer, Corey N. Miller, Jessica Chavez, David I. Berrios, Lauren E. Byrnes, Joe Germino, Vasilis Ntranos, Julie B. Sneddon, Trevor D. Burt, James M. Gardner, Chun J. Ye, Mark S. Anderson, Audrey V. Parent

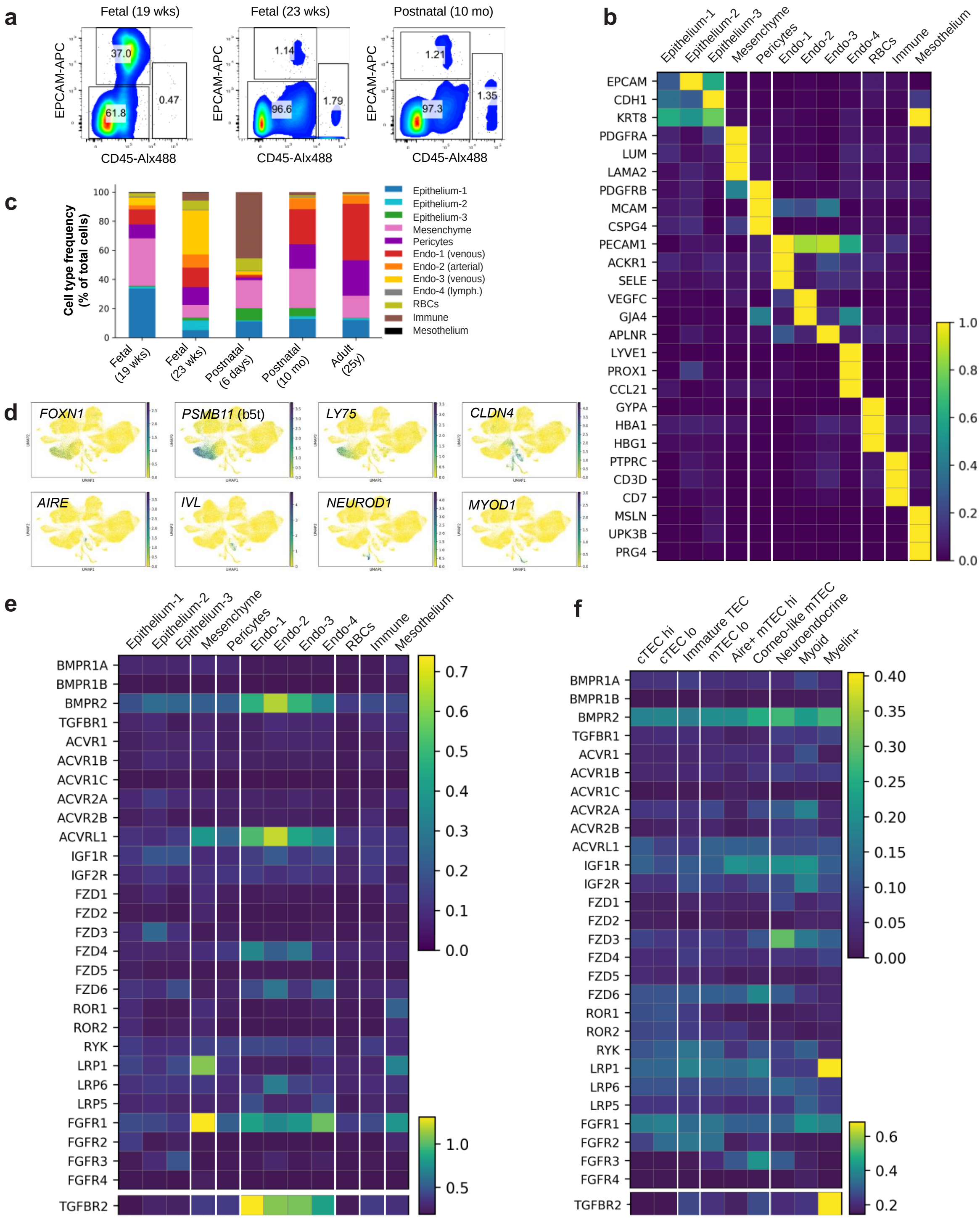

Supplementary Figure 1. a. Flow cytometric analysis of single cell preparations after digestion and enrichment of stromal cells. **b**. Heatmap showing the expression of marker genes for each subset of cells. **c**. Stacked bar graph of cell type frequency in each sample. Source data are provided as a Source Data file. **d**. UMAP visualization of markers for TECs and other epithelial subsets. **e-f**. Heatmap showing the expression of WNT, BMP, TGF beta, IGF, and FGF receptors in all subpopulations (**e**) or in TEC subsets (**f**).

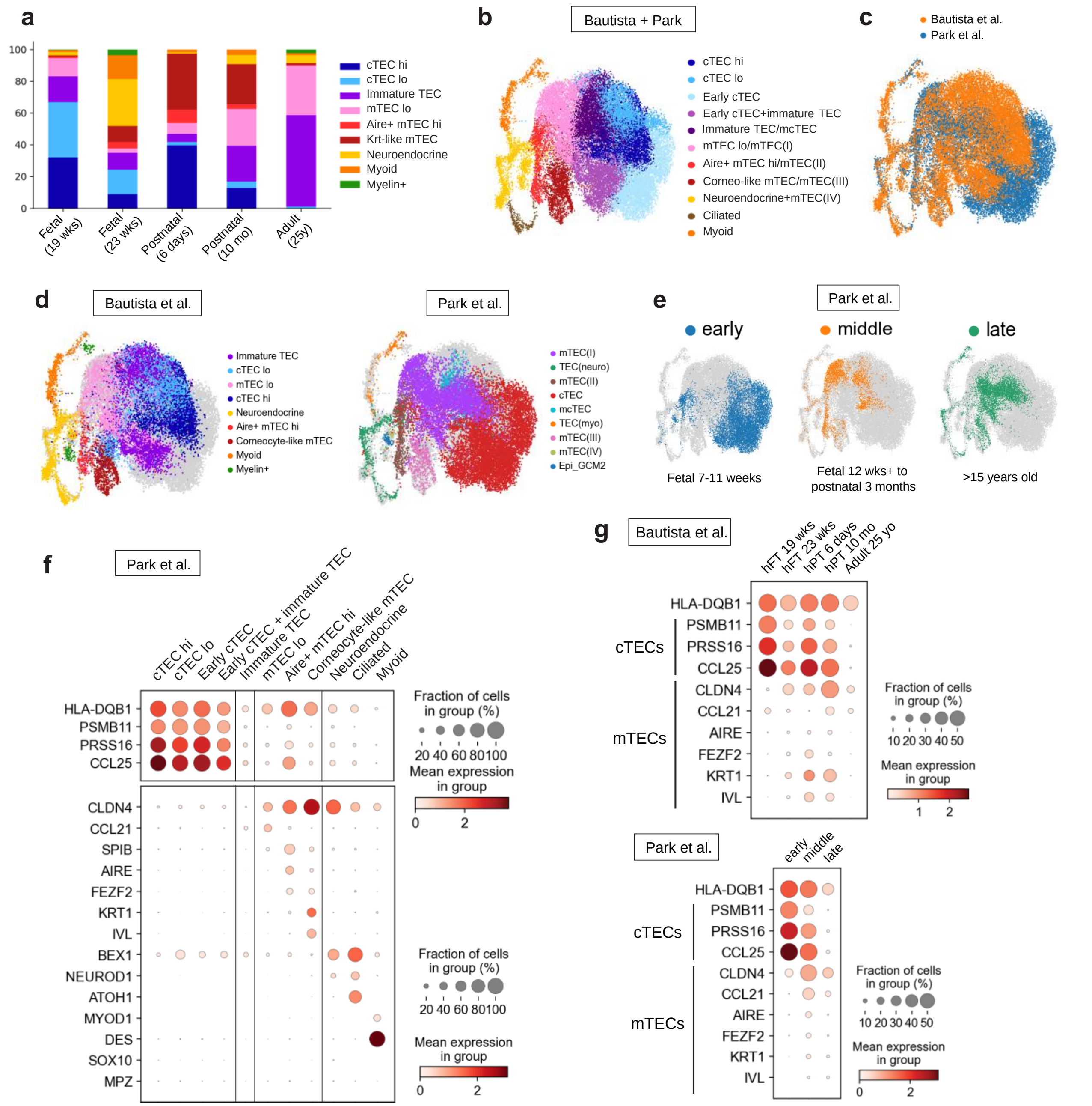

Supplementary Figure 2. a. Stacked bar graph of epithelial subset frequency in each sample. Source data are provided as a Source Data file. **b-c**. UMAP visualization of epithelial subsets from the combined Bautista + Park dataset colored by cell type (**b**) or source (**c**). UMAP visualization of epithelial subsets from this study (Bautista et al.) or the Park et al. study. **d.** UMAP visualization of the Bautista and Park et al. subsets colored by cell type. **e**. UMAP visualization of the Park et al. subsets colored by stage (early: fetal 7-11 weeks, middle: fetal 12 wks+ to postnatal 3 months, late: more than 15 years old). **f**. Dot plot showing the expression of known marker genes in the Park et al. dataset. **g**. Dot plot depicting the expression of cTEC and mTEC markers in human fetal thymus (hFT), human postnatal thymus (hPT) or adult thymus in both datasets.

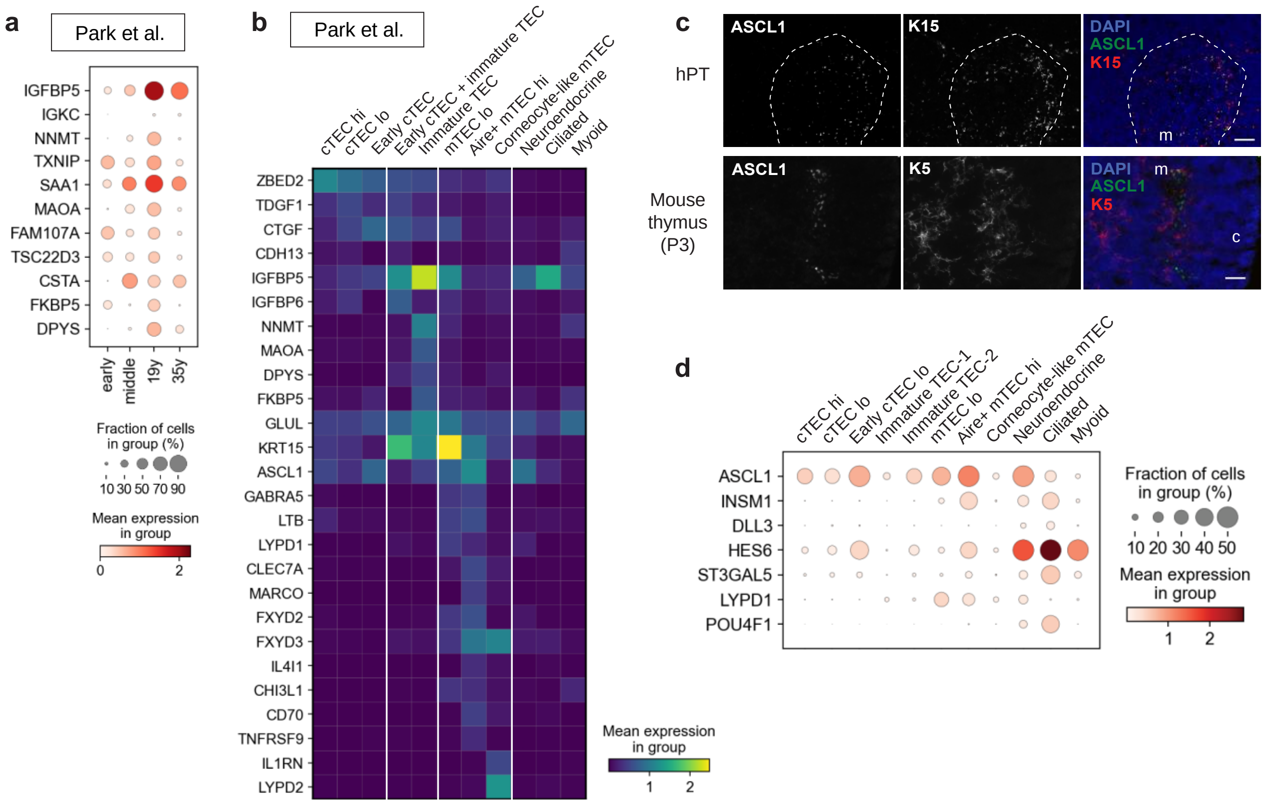

Supplementary Figure 3. a. Dot plot showing immature TEC gene expression across age groups in the Park et al. dataset. **b**. Heatmap of the expression of new TEC markers in the Park et al. dataset. **c**. Immunofluorescence analysis of ASCL1 in human postnatal thymus (hPT) and mouse neonatal thymus indicating that its expression is restricted to the medulla. Scale bars, 100 μm (top panel) and 50 μm (lower panel). Staining was repeated three times with similar results. **d**. Dot plot showing the expression of ASCL1 target genes in epithelial subsets of the combined Bautista + Park dataset.

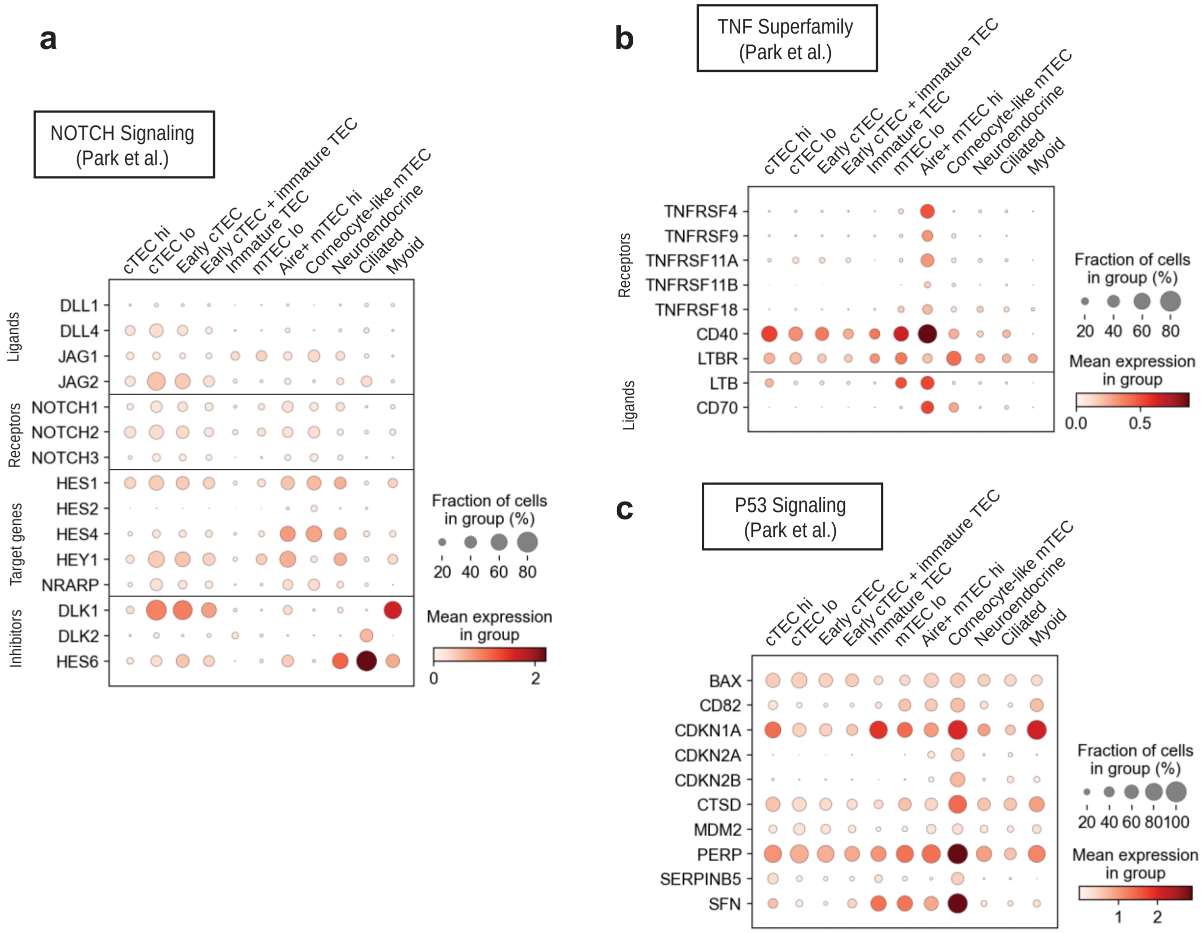

Supplementary Figure 4. a. Dot plot depicting the relative level of expression of Notch signaling ligands, receptors, target genes, and inhibitors in epithelial subsets from the Park et al. dataset. **b-c**. Dot plots depicting the relative level of expression of selected TNF Superfamily (**b**) or p53 signaling pathway (**c**) genes in the Park et al. dataset.

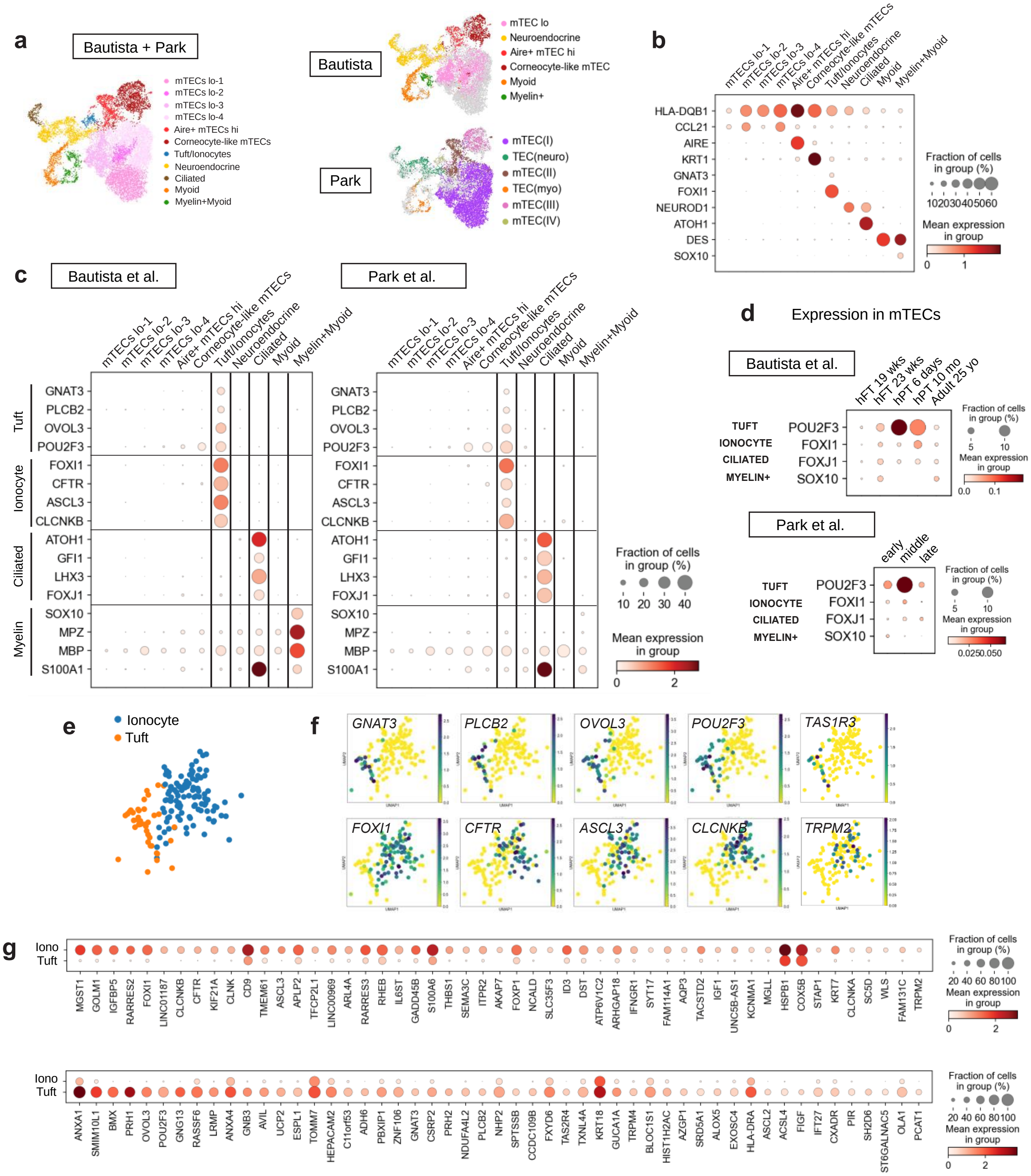

Supplementary Figure 5. a. UMAP visualization of Bautista + Park combined medullary epithelial subsets colored by cell type. Epithelial subsets from this study (Bautista et al.) or the Park et al. study are also shown. **b**. Dot plot showing the expression of known marker genes in the combined dataset. **c**. Dot plot showing the expression of tuft, ionocytes, ciliated, and myelin-expressing markers in each dataset. **d**. Dot plot showing the expression of tuft, ionocytes, ciliated, and myelin-expressing markers in each age group. **e**. UMAP visualization of the tuft/ionocyte cluster colored by cell type. **f**. UMAP visualization of tuft and ionocytes marker genes. **g**. Dot plots showing the expression of newly identified tuft and ionocytes markers in each subset.

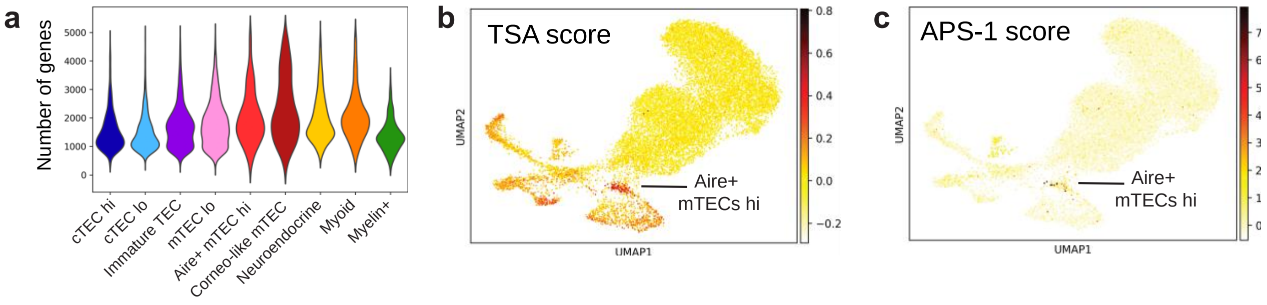

Supplementary Figure 6. a. Violin plot depicting the number of genes per cell for each epithelial cluster. **b-c**. UMAP visualization of the average expression of tissue-specific antigens (TSA score) (**b**) or antigens eliciting auto-antibodies in APS-1 patients (**c**) in epithelial cells.

Supplementary Table 1. List of primary antibodies used in this study.

Antibody	Manufacturer	Catalog number	Dilution
EPCAM APC	BioLegend	324208	1:100
CD45 Alx488	BioLegend	304017	1:100
CD11c PE-Cy7	BioLegend	117318	1:200
CD45 PerCP	BioLegend	103130	1:200
EpCAM APC-Cy7	BioLegend	118218	1:200
I-Ab eFluor450	eBiosciences	48-5320-82	1:200
KRT8-Alx488	Abcam	ab192467	1:500
KRT8-Alx647	Abcam	ab192468	1:500
KRT5-Alx488	Abcam	ab193894	1:500
KRT5-Alx647	Abcam	ab193895	1:500
KRT15-Alx555	Abcam	ab214393	1:500
KRT10-Alx647	Abcam	ab194231	1:500
KRT5	Abcam	ab53121	1:500
KRT15	Abcam	ab80522	1:200
Fibronectin	Sigma	F3648	1:200
CFTR	R&D Systems	MAB25031	1:100
DESMIN	Dako	M0760	1:100
Synaptophysin	Biogenex	AM363	1:100
ASCL1	Abcam	ab74065	1:100
AIRE	eBiosciences	14-9534-82	1:100
SOX2	R&D Systems	AF2018	1:100
wide spectrum cytokeratin	Abcam	ab9377	1:200
CD8-Alx488	BioLegend	300916	1:50
CD4-Alx647	BioLegend	300520	1:50
CDH13	R&D Systems	AF3264	1:150
TRPM2	Alomone labs	ACC-043	1:100
HES1	Abcam	ab108937	1:300
