# Supplementary material for: Single-cell transcriptional profiling of human thymic stroma uncovers novel cellular heterogeneity in the thymic medulla
Source: Nat Commun. 2021 Feb 17;12:1096. doi: 10.1038/s41467-021-21346-6 (PMC7889611; doi:10.1038/s41467-021-21346-6)
Supplement: Supplementary file 3 — Reporting Summary [file 41467_2021_21346_MOESM3_ESM.pdf]

nature research

Corresponding author(s): Audrey Parent

Last updated by author(s): Jan 18, 2021

Reporting Summary

Nature Research wishes to improve the reproducibility of the work that we publish. This form provides structure for consistency and transparency in reporting. For further information on Nature Research policies, see our Editorial Policies and the Editorial Policy Checklist.

Statistics

For all statistical analyses, confirm that the following items are present in the figure legend, table legend, main text, or Methods section.

n/a	Confirmed	
[ ]	[x]	The exact sample size (*n*) for each experimental group/condition, given as a discrete number and unit of measurement
[ ]	[x]	A statement on whether measurements were taken from distinct samples or whether the same sample was measured repeatedly
[ ]	[x]	The statistical test(s) used AND whether they are one- or two-sided<br>*Only common tests should be described solely by name; describe more complex techniques in the Methods section.*
[ ]	[x]	A description of all covariates tested
[ ]	[x]	A description of any assumptions or corrections, such as tests of normality and adjustment for multiple comparisons
[ ]	[x]	A full description of the statistical parameters including central tendency (e.g. means) or other basic estimates (e.g. regression coefficient) AND variation (e.g. standard deviation) or associated estimates of uncertainty (e.g. confidence intervals)
[ ]	[x]	For null hypothesis testing, the test statistic (e.g. *F*, *t*, *r*) with confidence intervals, effect sizes, degrees of freedom and *P* value noted<br>*Give P values as exact values whenever suitable.*
[x]	[ ]	For Bayesian analysis, information on the choice of priors and Markov chain Monte Carlo settings
[x]	[ ]	For hierarchical and complex designs, identification of the appropriate level for tests and full reporting of outcomes
[ ]	[x]	Estimates of effect sizes (e.g. Cohen's *d*, Pearson's *r*), indicating how they were calculated

Our web collection on statistics for biologists contains articles on many of the points above.

Software and code

Policy information about availability of computer code

Data collection	Flow Cytometry: BD FACSDiva v8.0.1
Data analysis	Graphad Prism8<br>TreeStar FlowJo v10.7.1<br>Microsoft Excel v.16.30<br>Cell Ranger/STAR software 2.0.0, 2.1.1, or 3.0.2<br>Scanpy v1.4.4 or 1.6.0<br>scVelo 0.1.25

For manuscripts utilizing custom algorithms or software that are central to the research but not yet described in published literature, software must be made available to editors and reviewers. We strongly encourage code deposition in a community repository (e.g. GitHub). See the Nature Research guidelines for submitting code & software for further information.

Data

Policy information about availability of data

All manuscripts must include a data availability statement. This statement should provide the following information, where applicable:

- Accession codes, unique identifiers, or web links for publicly available datasets
- A list of figures that have associated raw data
- A description of any restrictions on data availability

Data for the single-cell RNA sequencing have been deposited in the Gene Expression Omnibus (GEO) database under accession number GSE147520 (https://www.ncbi.nlm.nih.gov/geo/query/acc.cgi?acc=GSE147520). The Park et al. dataset was downloaded from the Zenodo repository (DOI: 10.5281/zenodo.3711134).

Field-specific reporting

Please select the one below that is the best fit for your research. If you are not sure, read the appropriate sections before making your selection.

☒ Life sciences ☐ Behavioural & social sciences ☐ Ecological, evolutionary & environmental sciences

For a reference copy of the document with all sections, see nature.com/documents/nr-reporting-summary-flat.pdf

Life sciences study design

All studies must disclose on these points even when the disclosure is negative.

Sample size	For single-cell RNA sequencing, five donors were chosen to represent donor variability, which was sufficient to define broad cell populations representative of the human thymic stroma. The majority of our analyses had hundreds to thousands of cells for each cell type, which allowed identification of many statistically significant differentially expressed genes. In addition, we increased our sample size by merging our dataset with the publicly available dataset from the Park et al. study (DOI: 10.1126/science.aay3224). For immunofluorescence imaging, representative images from 4-5 fields from at least two donors were shown. For the lineage tracing experiment, no statistical methods were used to predetermine sample size. Sample size was chosen based on the magnitude and consistency of measurable differences between groups and application of standard practices within the field.
Data exclusions	Computational cell and gene pre-filtering of single cell data was described in the Methods section. Cells with less than 200 or more than 5000 detected genes were excluded as well as cells expressing >10% mitochondrial genes.
Replication	Single-cell RNA sequencing was performed using cells from five donors. Merging our dataset with the Park et al. dataset containing cells from twelve donors largely replicated our findings. All other experimental findings described in this manuscript were also reliably reproduced between different donors.
Randomization	Animals were age-matched and were allocated to groups based on genotype. Whenever possible, animals were co-housed with littermate controls and sex-matched.
Blinding	Researchers were not blinded during experiments because our study results did not involve the combination of subjective decisions or a priori hypotheses, where classical blinding might be needed. Rather, we conducted computational analyses and genome-wide statistical tests, then reported the results of these agnostic analyses. Our findings were also supported by quantitative measure (i.e. flow cytometry) where blinding was unnecessary. Finally, investigators were not blinded to mouse genotypes in our experiments. This was to help ensure that appropriate sample size was achieved by sacrificing the minimum number of mice required to achieve consistent data.

Reporting for specific materials, systems and methods

We require information from authors about some types of materials, experimental systems and methods used in many studies. Here, indicate whether each material, system or method listed is relevant to your study. If you are not sure if a list item applies to your research, read the appropriate section before selecting a response.

Materials & experimental systems

n/a	Involved in the study	
☐	☒	Antibodies
☒	☐	Eukaryotic cell lines
☒	☐	Palaeontology and archaeology
☐	☒	Animals and other organisms
☐	☒	Human research participants
☒	☐	Clinical data
☒	☐	Dual use research of concern

Methods

n/a	Involved in the study	
☒	☐	ChIP-seq
☐	☒	Flow cytometry
☒	☐	MRI-based neuroimaging

Antibodies

Antibodies used	EPCAM APC BioLegend 324208<br>CD45 Alx488 BioLegend 304017<br>CD11c PE-Cy7 BioLegend 117318<br>CD45 PerCP BioLegend 103130<br>EpCAM APC-Cy7 BioLegend 118218<br>I-Ab eFluor450 eBiosciences 48-5320-82<br>KRT8-Alx488 Abcam ab192467<br>KRT8-Alx647 Abcam ab192468<br>KRT5-Alx488 Abcam ab193894<br>KRT5-Alx647 Abcam ab193895<br>KRT15-Alx555 Abcam ab214393<br>KRT10-Alx647 Abcam ab194231<br>KRT5 Abcam ab53121<br>KRT15 Abcam ab80522<br>Fibronectin Sigma F3648<br>CFTR R&D Systems MAB25031<br>DESMIN Dako M0760<br>Synaptophysin Biogenex AM363<br>ASCL1 Abcam ab74065<br>AIRE eBiosciences 14-9534-82<br>SOX2 R&D Systems AF2018<br>wide spectrum cytokeratin Abcam ab9377<br>CD8-Alx488 BioLegend 300916<br>CD4-Alx647 BioLegend 300520<br>CDH13 R&D Systems AF3264<br>TRPM2 Alomone labs ACC-043<br>HES1 Abcam ab108937
Validation	All primary antibodies were validated by the manufacturer and/or in previously published articles.<br><br>CD11c PE-Cy7 (Clone N418, 117318, BioLegend), CD45 PerCP (Clone 30-F11, 103130, BioLegend), EpCAM APC-Cy7 (Clone G8.8, 118218, BioLegend) and I-Ab eFluor450 (Clone 25-9-17, 48-5320-82, eBiosciences) were validated on mouse thymic tissue in a previous study (Miller et al., Nature 559, 627–631 (2018).<br><br>The specificity of primary antibodies for immunofluorescent staining was verified on human thymic sections using various dilutions (including the manufacturer's recommended dilution). Secondary antibody only controls were used to evaluate and confirm specificity of the antibodies to the respective epitopes. The following antibodies used for flow cytometry were similarly validated on human thymic preparations:<br>EPCAM APC, BioLegend, 324208<br>CD45 Alx488, BioLegend, 304017<br>KRT8-Alx488, Abcam, ab192467<br>KRT5-Alx647 Abcam ab193895<br>KRT15-Alx555 Abcam ab214393

Animals and other organisms

Policy information about studies involving animals; ARRIVE guidelines recommended for reporting animal research

Laboratory animals	Rosa26CAG-stopflox-tdTomato and Ascl1-creERT2 mice were obtained from The Jackson Laboratory (RRID:IMSR_JAX:007914 and RRID:IMSR_JAX:012882). ADIG mice have been described previously (DOI: 10.1126/science.1159407). Both male and female mice aged 12-15 weeks were used. Mice were maintained at a constant humidity between 30-70% and temperature 68-79 degrees Fahrenheit, under a 12-h light/dark cycle and had free access to food and water.
Wild animals	The study did not involve wild animals.
Field-collected samples	Field samples were not collected for the study.
Ethics oversight	UCSF Institutional Animal Care and Use Committee (IACUC) and Laboratory Animal Resource Center

Note that full information on the approval of the study protocol must also be provided in the manuscript.

Human research participants

Policy information about studies involving human research participants

Population characteristics	Thymic tissue donors were male and female between 19 weeks gestational age and 25 years old. Tissue was de-identified at the point of collection.

Recruitment	Pediatric tissues were obtained from patients undergoing corrective cardiothoracic surgery. Human adult thymic tissues were acquired from research-consented deceased organ donors at the time of organ acquisition for clinical transplantation. Human fetal thymus was obtained from 19- to 23-gestational-week specimens under the guidelines of the University of California San Francisco Committee on Human Research.
Ethics oversight	Pediatric tissues were obtained from patients undergoing corrective cardiothoracic surgery in accordance with protocols approved by the UCSF Human Research Protection Program Institutional Review Board (IRB #17-22928). Written informed consent was obtained from the patient's parents, guardians or Legally Authorized Representatives (LARs) before sample collection. Human adult thymic tissues were acquired from research-consented deceased organ donors at the time of organ acquisition for clinical transplantation through an IRB-approved research protocol with Donor Network West, the organ procurement organization for Northern California. Human fetal thymus was obtained from 19- to 23-gestational-week specimens under the guidelines of the University of California San Francisco Committee on Human Research.

Note that full information on the approval of the study protocol must also be provided in the manuscript.

Flow Cytometry

Plots

Confirm that:

☒ The axis labels state the marker and fluorochrome used (e.g. CD4-FITC).

☒ The axis scales are clearly visible. Include numbers along axes only for bottom left plot of group (a 'group' is an analysis of identical markers).

☒ All plots are contour plots with outliers or pseudocolor plots.

☒ A numerical value for number of cells or percentage (with statistics) is provided.

Methodology

Sample preparation	Thymic tissues placed in RPMI (ThermoFisher) containing 100 µg/ml DNase I (Roche) were cut into small pieces using scissors. Tissue pieces were transferred into a gentleMACS C tube (Miltenyi) containing 10 ml of RPMI with DNAse. The gentleMACS Program m_spleen_02 was run three times. Thymic fragments were separated from the thymocytes-rich supernatant by centrifugation. Remaining fragments were transferred back to C tube with fresh RPMI with DNAse before running program m_spleen_01. The supernatant was removed and replaced with 10 ml of digestion medium containing 100 µg/ml DNase I and 100 µg/ml Liberase TM (Sigma-Aldrich) in RPMI. Tubes were moved to a 37°C water bath and fragments were triturated every 5 minutes to mechanically aid digestion. At 30 min, tubes were spun briefly to pellet undigested fragments and the supernatant was discarded. Fresh digestion medium or accumax (STEMCELL Technologies) was added to remaining fragments and the digestion was repeated for another 15-30 minutes until most pieces were digested. Supernatant from this second round of digestion was transferred to a tube containing cold MACS buffer (0.5% BSA, 2 mM EDTA in PBS) to stop the enzymatic digestion. If necessary, a third round of enzymatic digestion was performed on remaining fragments using accumax for an additional 5-10 min. Cells were pooled with the supernatant from the previous round of digestion and were passed through a 40 µm filter (Falcon). Some samples were treated with 2 ml of ACK lysing buffer (Lonza) for 5 min prior to stromal enrichment.
Instrument	Data were collected using BD LSR2, BD Fortessa, and BD Aria Fusion instruments.
Software	Flow cytometry data were collected and analyzed using the BD FACSDiva and TreeStar FlowJo softwares.
Cell population abundance	Cell purity following sorting was not assessed.
Gating strategy	All cells were gated on singlets, live cells, and FSC/SSC.<br>Murine TECs: CD11c- CD45- EPCAM+<br>Human TECs: CD45- EPCAM+

☒ Tick this box to confirm that a figure exemplifying the gating strategy is provided in the Supplementary Information.
