# Supplementary material for: Single-cell transcriptional profiling of human thymic stroma uncovers novel cellular heterogeneity in the thymic medulla
Source: Nat Commun. 2021 Feb 17;12:1096. doi: 10.1038/s41467-021-21346-6 (PMC7889611; doi:10.1038/s41467-021-21346-6)
Supplement: Supplementary file 4 — Description of Additional Supplementary Files [file 41467_2021_21346_MOESM4_ESM.pdf]

Description of Additional Supplementary Files

Supplementary Data 1. Marker genes for all stromal cell clusters. Marker genes were identified by testing for differential expression of a subgroup against all other cells using a t-test with overestimated variance implemented in the tl.rank_genes_groups function of Scanpy.

Supplementary Data 2. Marker genes for epithelial clusters. Marker genes were identified by testing for differential expression of a subgroup against all other cells using a t-test with overestimated variance implemented in the tl.rank_genes_groups function of Scanpy.

Supplementary Data 3. Marker genes for immature TECs. Marker genes were identified by testing for differential expression of a subgroup against all other cells using a t-test with overestimated variance implemented in the tl.rank_genes_groups function of Scanpy.

Supplementary Data 4. Marker genes for medullary epithelial clusters. Marker genes were identified by testing for differential expression of a subgroup against all other cells using a t-test with overestimated variance implemented in the tl.rank_genes_groups function of Scanpy.

Supplementary Data 5. Genes differentially expressed in tuft cells vs ionocytes. Characteristic gene signatures were identified by testing for differential expression of a subgroup against all other cells using a t-test with overestimated variance implemented in the tl.rank_genes_groups function of Scanpy.

Supplementary Data 6. Genes with expression that positively correlate with a high TSA score in AIRE+ and corneocyte-like mTECs. A Spearman correlation coefficient with associated p-value was calculated using the spearmanr function from the SciPy statistical package. The list was filtered to keep genes expressed in at least five cells that had positive correlation coefficients >0.15 and p values < 0.25.
